# Methicillin-resistant Staphylococcus Aureus Colitis Secondary to Complicated Acute Diverticulitis: A Rare Case Report

**DOI:** 10.7759/cureus.5013

**Published:** 2019-06-27

**Authors:** Everett Rogers, Allison Dooley, Samantha Vu, Furqan Haq, Spiros Ferderigos

**Affiliations:** 1 Internal Medicine, Nova Southeastern University's Dr. Kiran C. Patel College of Osteopathic Medicine, Davie, USA; 2 Internal Medicine, Largo Medical Center, Largo, USA; 3 Psychiatry, Largo Medical Center, Largo, USA; 4 Internal Medicine, Oak Hill Hospital, Tampa, USA

**Keywords:** mrsa colitis, diverticulitis, clostridium difficile

## Abstract

A 33-year-old obese female with a recent diagnosis of acute diverticulitis (AD) was admitted to the hospital for severe abdominal pain, intractable nausea and vomiting, and diarrhea two days following oral antibiotic treatment for AD. Stool cultures collected upon her readmission were negative for Clostridium difficile (C. difficile) antigen and toxins A and B, but were notable for methicillin-resistant Staphylococcus aureus (MRSA). She was started on intravenous (IV) piperacillin/tazobactam, IV vancomycin, and an oral liquid vancomycin solution, which resulted in rapid resolution of her symptoms. Unfortunately, her symptoms recurred two weeks later and she eventually underwent laparoscopic low anterior resection (LAR) of her colon for continued diverticulitis. This resulted in complete and continued resolution of her symptoms.

## Introduction

Prior to the implication of toxin-producing clostridia as the main etiologic agent of antibiotic-associated colitis in 1978, Staphylococcus aureus (S. aureus) was widely believed to play a major role in the pathogenesis of this disease [1]. Today, S. aureus is rarely, if ever, included in a physician’s differential diagnosis as the causative agent of antibiotic-associated colitis, which may therefore lead to it being widely underdiagnosed. Although Clostridium difficile (C. difficile) is the most common cause of antibiotic-associated diarrhea, it only accounts for 15%-25% of cases [2]. Here we present an unusual case of complicated methicillin-resistant Staphylococcus aureus (MRSA) colitis possibly resulting from antibiotic treatment in the setting of diverticulitis.

## Case presentation

A 33-year-old obese female with a past medical history of uterine cancer status-post hysterectomy and chemotherapy, mild beta thalassemia, and who works as a nurse at the local county jail, was admitted to the hospital complaining of severe abdominal pain and intractable nausea, vomiting, and diarrhea for the past five days. Ten days prior, she had been admitted to the hospital for severe sepsis secondary to acute diverticulitis (AD), which was confirmed with computed tomography (CT) of the abdomen. During the same admission, she was found to have impaired fasting blood glucose and non-alcoholic fatty liver disease. After proper treatment leading to resolution of her symptoms, she was subsequently discharged and instructed to finish the eight-day course of metronidazole and ciprofloxacin that had been started, and to follow up with gastroenterology for colonoscopy and evaluation of diverticulitis.

**Figure 1:**
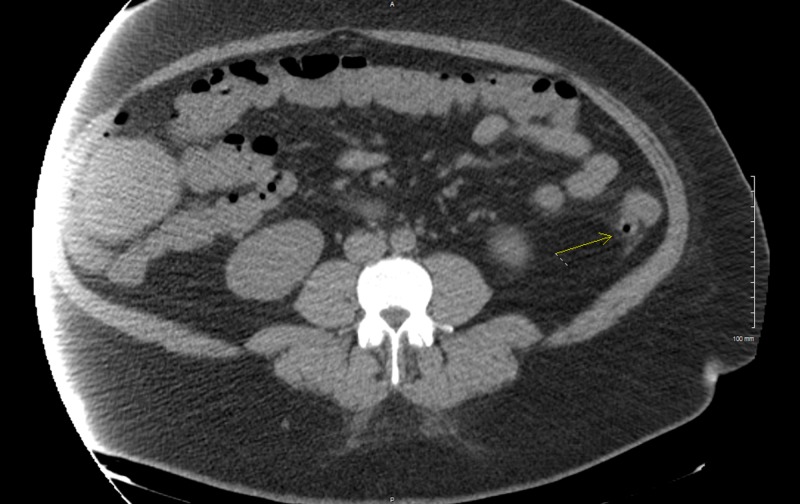
Computed tomography (CT) scan showing diverticula (yellow arrow) in the descending colon with fat stranding

On examination during her return admission, the patient was afebrile with a temperature of 99.3^o^F, heart rate of 103 beats per minute, respiratory rate of 18 breathes per minute, and blood pressure of 150/88 mmHg. She also had diffuse abdominal tenderness. Labs revealed a white blood cell count of 14.2 k/uL and a qualitative stool lactoferrin test was positive. Repeat CT abdomen and pelvis showed mild diverticulitis of the mid and distal descending colon, unchanged since her last visit (Figure 1). Stool cultures were negative for Salmonella, Shigella, Aeromonas, or Pleisiomonas. However, a predominance of MRSA was cultured. Studies for C. difficile antigen and toxins A and B were negative. Fecal occult blood test was positive.

There was initial debate as to whether or not the MRSA in the stool culture was a true infection or just colonization. However, after a review of current literature, and discussion with two infectious disease specialists and a gastroenterologist, the patient was started on a 14-day course of intravenous (IV) piperacillin/tazobactam, IV vancomycin, and an oral liquid vancomycin solution. Antiemetics and analgesics were administered concurrently, and the patient experienced a rapid resolution of her symptoms with normalization of her infectious laboratory markers. 

At the end of the 14 days of treatment, her abdominal pain returned and she presented once again to the hospital. Repeat CT scan of the abdomen during this visit was consistent with unresolved diverticulitis. Gastroenterology and general surgery were consulted, and stool cultures were repeated which no longer grew any pathologic organisms, including MRSA. After consultation with general surgery, a decision was made to proceed with a low anterior resection (LAR). Post-operatively, the pathology of the resected colon showed numerous diverticula with lymphoid aggregates and chronic inflammation. She recovered well post-operatively and was discharged home on post-op day six without any further need for antibiotic therapy. She returned to the emergency room (ER) 16 days later with abdominal wound dehiscence and dark brown drainage from the abdomen, which grew MRSA on wound cultures. The patient was discharged from the ER to follow up with surgery outpatient.

## Discussion

Before the seminal paper written by Bartlett et al. in 1978 that implicated C. difficile as the main etiologic agent of antibiotic-associated diarrhea, S. aureus was generally believed to play a major pathophysiologic role in the development of the disease [1]. While C. difficile has since gained near-universal recognition as the cause of antibiotic-associated diarrhea, enterotoxin-producing strains of S. aureus should not be entirely dismissed as a possible culprit in certain cases [3-5].

In a study by Gravet et al., S. aureus was isolated as the predominate or only isolate from a cohort of 60 hospital patients, 90% of whom had diarrhea, and 98% of whom had received antibiotics within the last month [3]. Another study conducted by Boyce and Havill found that of 3,590 stool screenings performed at one university hospital, 321 (8.9%) yielded MRSA. Of those positive for MRSA, 13 were negative for C. difficile and met the criteria for antibiotic-associated diarrhea due to enterotoxin-producing MRSA, which included ruling out other bacterial enteric pathogens, ova and parasites, and viruses [5]. These studies emphasize the importance of keeping enterotoxin-producing MRSA species on one’s clinical differential when attempting to determine the cause, and treatment, of C. difficile-negative antibiotic-associated colitis.

Furthermore, the predominant growth of MRSA in stool cultures is not typically flagged as an abnormal result, and could therefore have been easily overlooked in this case. This raises a question of the incidence of MRSA-predominant stool culture growth, as it may often be overlooked as a “normal” result. This fact is especially poignant considering that C. difficile is the culprit in only 15%-25% of cases of antibiotic-associated diarrhea [2].

This case illustrates a rare occurrence of diverticulitis which was complicated by antibiotic-associated MRSA colitis. A small handful of case reports have been published over the past few years of patients suffering from symptomatic colitis in whom stool cultures resulted in heavy growth of MRSA following a treatment course of antibiotics [4,6-10]. Our patient’s case differs from prior case reports in that rather than just colitis, our patient had severe refractory diverticulitis which initially improved with standard diverticulitis treatment. However, it recurred after a full antibiotic course of ciprofloxacin and metronidazole. Stool cultures predominantly grew MRSA, which is outside the coverage of these antibiotics.

Considering the patient’s symptomatic improvement during a course of metronidazole and ciprofloxacin, the negative C. difficile toxin assay, and the predominance of MRSA on stool culture, it is reasonable to consider that this was a case of diverticulitis complicated by antibiotic-associated mid-distal colitis due to enterotoxin-producing MRSA. In conjunction with the results of our literature review, this case supports the need for a paradigm shift in our clinical understanding and treatment of antibiotic-associated colitis in the future. Enterotoxin-producing MRSA strains as a cause of antibiotic-associated colitis is a real and likely growing problem, and should therefore be kept on the clinician’s list of differential diagnoses when managing the care of these patients [3-10].

## Conclusions

Antibiotic-associated colitis is a relatively common occurrence. Although it is usually due to overgrowth of C. difficile, a review of current literature demonstrates that MRSA is likely an under-reported cause. MRSA should be considered as a possible etiology if stool antigen assays for more common causes of antibiotic-associated bacteria, including C. difficile, are negative, while cultures grow a predominance of MRSA. Suspicion should also be raised if a patient belongs to a high-risk demographic, like healthcare workers, as was the case with this patient.
